# Supplementary figures and images for: Intraspecific genetic variability and diurnal activity affect environmental DNA detection in Japanese eel
Source: PLoS One. 2021 Sep 16;16(9):e0255576. doi: 10.1371/journal.pone.0255576 (PMC8445453; doi:10.1371/journal.pone.0255576)

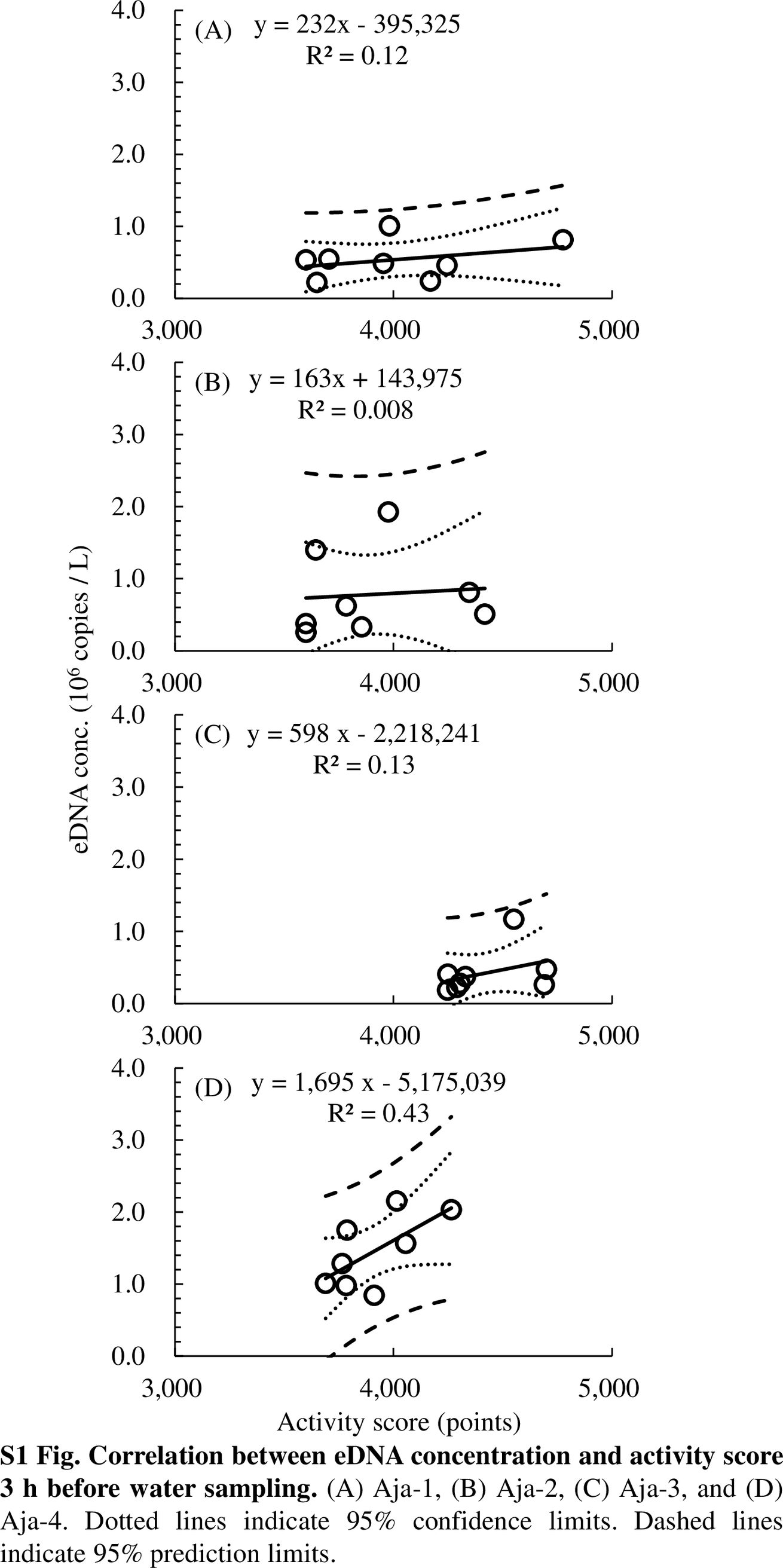

Supplement: S1 Fig — (TIF) [file pone.0255576.s001.tif]
