# Supplementary material for: Intraspecific genetic variability and diurnal activity affect environmental DNA detection in Japanese eel
Source: PLoS One. 2021 Sep 16;16(9):e0255576. doi: 10.1371/journal.pone.0255576 (PMC8445453; doi:10.1371/journal.pone.0255576)
Supplement: Attachment — Submitted filename: Response to Reviewers.docx [file pone.0255576.s011.docx]

***Response and our reply were shown in boldfaced type.**

PONE-D-20-38269R1

Intraspecific genetic variability and diurnal activity affect environmental DNA detection in Japanese eel

PLOS ONE

Dear Dr. Takahashi,

Thank you for submitting your manuscript to PLOS ONE. After careful consideration, we feel that it has merit but does not fully meet PLOS ONE’s publication criteria as it currently stands. Therefore, we invite you to submit a revised version of the manuscript that addresses the points raised during the review process.

==============================

I got the recommendations and comments from a previous reviewers. The reviewer agree that the manuscript is improved but suggested that more experimental work and careful discussion are necessary for examining the effect of mismatch between probe and target sequence on detection efficiency and provided other major/minor comments. I totally share these comments. Therefore, I can invite you to submit a revised version of the manuscript that addresses the points raised by the reviewer.

==============================

Please submit your revised manuscript by Jul 01 2021 11:59PM. If you will need more time than this to complete your revisions, please reply to this message or contact the journal office at plosone@plos.org. When you're ready to submit your revision, log on to https://www.editorialmanager.com/pone/ and select the 'Submissions Needing Revision' folder to locate your manuscript file.

Please include the following items when submitting your revised manuscript:

A rebuttal letter that responds to each point raised by the academic editor and reviewer(s). You should upload this letter as a separate file labeled 'Response to Reviewers'.

A marked-up copy of your manuscript that highlights changes made to the original version. You should upload this as a separate file labeled 'Revised Manuscript with Track Changes'.

An unmarked version of your revised paper without tracked changes. You should upload this as a separate file labeled 'Manuscript'.

If you would like to make changes to your financial disclosure, please include your updated statement in your cover letter. Guidelines for resubmitting your figure files are available below the reviewer comments at the end of this letter.

If applicable, we recommend that you deposit your laboratory protocols in protocols.io to enhance the reproducibility of your results. Protocols.io assigns your protocol its own identifier (DOI) so that it can be cited independently in the future. For instructions see: http://journals.plos.org/plosone/s/submission-guidelines#loc-laboratory-protocols. Additionally, PLOS ONE offers an option for publishing peer-reviewed Lab Protocol articles, which describe protocols hosted on protocols.io. Read more information on sharing protocols at https://plos.org/protocols?utm_medium=editorial-email&utm_source=authorletters&utm_campaign=protocols.

We look forward to receiving your revised manuscript.

Kind regards,

Hideyuki Doi

Academic Editor

PLOS ONE

Additional Editor Comments (if provided):

I got the recommendations and comments from a previous reviewers. The reviewer agree that the manuscript is improved but suggested that more experimental work and careful discussion are necessary for examining the effect of mismatch between probe and target sequence on detection efficiency and provided other major/minor comments. I totally share these comments. Therefore, I can invite you to submit a revised version of the manuscript that addresses the points raised by the reviewer.

[Note: HTML markup is below. Please do not edit.]

Reviewers' comments:

Reviewer's Responses to Questions

Comments to the Author

1. If the authors have adequately addressed your comments raised in a previous round of review and you feel that this manuscript is now acceptable for publication, you may indicate that here to bypass the “Comments to the Author” section, enter your conflict of interest statement in the “Confidential to Editor” section, and submit your "Accept" recommendation.

Reviewer #1: (No Response)

2. Is the manuscript technically sound, and do the data support the conclusions?

The manuscript must describe a technically sound piece of scientific research with data that supports the conclusions. Experiments must have been conducted rigorously, with appropriate controls, replication, and sample sizes. The conclusions must be drawn appropriately based on the data presented.

Reviewer #1: Partly

3. Has the statistical analysis been performed appropriately and rigorously?

Reviewer #1: I Don't Know

4. Have the authors made all data underlying the findings in their manuscript fully available?

The PLOS Data policy requires authors to make all data underlying the findings described in their manuscript fully available without restriction, with rare exception (please refer to the Data Availability Statement in the manuscript PDF file). The data should be provided as part of the manuscript or its supporting information, or deposited to a public repository. For example, in addition to summary statistics, the data points behind means, medians and variance measures should be available. If there are restrictions on publicly sharing data—e.g. participant privacy or use of data from a third party—those must be specified.

Reviewer #1: Yes

5. Is the manuscript presented in an intelligible fashion and written in standard English?

PLOS ONE does not copyedit accepted manuscripts, so the language in submitted articles must be clear, correct, and unambiguous. Any typographical or grammatical errors should be corrected at revision, so please note any specific errors here.

Reviewer #1: Yes

6. Review Comments to the Author

Please use the space provided to explain your answers to the questions above. You may also include additional comments for the author, including concerns about dual publication, research ethics, or publication ethics. (Please upload your review as an attachment if it exceeds 20,000 characters)

Reviewer #1: I have re-reviewed the manuscript “Intraspecific genetic variability and diurnal activity affect environmental DNA detection in Japanese eel” by Sayaka Takahashi et al. The revised manuscript addresses some of the major concerns; however, several more experimental work and careful discussion are necessary for examining the effect of mismatch between probe and target sequence on detection efficiency. I offer a few major and minor suggestions for improvement below.

**Our reply: We thank the reviewer for the constructive comments, which have substantially improve the readability of the manuscript.**

・The current title and text may cause the reader to misunderstand. This study suggested that mismatches between probes and target sequences, not genetic diversity, affect the efficiency of eDNA detection. In fact, SNP at a site other than the priming sites of primers and probes do not affect the estimation of eDNA concentration by qPCR.

**(Response)**

**We thank the reviewer for identifying the potential misunderstanding. The present result, i.e. less detection of eDNA in variants, indeed reflects the mismatches between probes and taget species as the reviewer suggested. However, essential finding of the study is that** **genetic variability would affect eDNA detection in the field even in a carefully designed set of primers and probe. We consider that there is a trade-off between speficity to target species and the risk of including variability in the probe region. This has been emphasized in the Discussion (Line 506–519) and we hope that our intention would be conveyed better in the revised manuscript.**

・In the responses to the reviewer, the authors used the same primers with qPCR for the Sanger sequencing to check the presence of SNPs. Again, “if the DNA amplicon was sequenced using the same primers as qPCR, you will not be able to find mismatches in the priming sites of the primers” because the priming site of the primers is replaced by the primer sequence. I think, this study did not examine whether there was a sequence mismatch in the primer positions (not probe). According to Kasai et al (2020), the priming sites of the primers sometimes have SNP. Thus, in my opinion, without confirming that there is no mismatch in the priming sites of the primers, the results in this study cannot properly understand and discussed.

**(Response)**

**Although the presence of SNPs affects DNA amplification, the DNA amplicon is certainly amplified in the following steps once the templates are annealed by the primers, because the sequence of DNA amplicon completely matches the sequence in the priming sites of the primers. On the other hand, mismatches of sequences between probe and templates are not eliminated in the following amplification steps. Therefore, we consider that the negative effect of polymorphs in probe region on DNA amplification should be substantial.**

**As per the reviewer pointed out, we could not discuss whether there was a sequence mismatch of the primer region in our study without samples. We cannot show the information that completely wipes out the concern of the reviewer. However, eDNA amplification in Variants by SYBR Green method was mach more larger than that by TaqMan method. DNAs of Vatiant-1 and Variant-2 were amplified to the same amount as Non variants’ by using the same primers with qPCR in additional experiment by SYBR Green method. Therefore, the mismatches in the priming sites of the primers are not likely to have affected eDNA detection.**

**Nevertheless, we agree that it is also important to consider such a possibility, and thus it has been mentioned as a key consideration in the revised Conclusions and perspectives section (****Line 608-611).**

・The overall impression is redundant because the point of the argument in each paragraph is not clear, and similar content is written several times.

**(Response)**

**We thank the reviewer for pointing out redundancy in the manuscript. We made efforts to streamline the text particularly in the Introduction section.**

L55-56: Itakura et al. (2019) demonstrated that eDNA analysis allows us to reveal the spatial distribution, abundance, and biomass of Japanese eels at the river-basin scale.

**(Response)**

**We are aware of the study of Itakura et al. (2019) as referred in the next paragraph of the former manuscript. Although their study surveying in more than 100 sites is impressive, the study not** **necesarily covered nation wide river basins. Besides, the topic in this first paragraph is the distribution of Japanese eel in general without a focus on eDNA. To avoid confusion we revised from “distribution” to “spatiotemporal distributions” and from “in rivers“ to “in various river sites“ in this sentence (Line 56-57).**

L73-75: Is this true? I think, the relationship between eDNA concentration and biomass and/or the number of individuals is a major topic that has been examined by a great number of studies, and some of which have reported a strong relationship.

**(Response)**

**Yes. It is true. Some researchers have reported a strong relationship between eDNA concentration and biomass and/or the number of individuals. However, other researchers, including Itakura et al. (2019), have reported only weak positive relationship between them (Line 82-83). Considering that non-significant results would be published less, we can say that the relationship between eDNA concentration and fish abundance is yet to be established.**

L79: “The eDNA concentration increases 10–200× during spawning [35].” → "[25]" ?

**(Response)**

**We thank the reviewer for pointing out this flaw. We revised the citation (Line 107).**

L90: Ghosal et al. (2018) reported that the carp eDNA concentration increased 500× at night when the fish biomass only doubled. However, this experiment was carried out under special experimental conditions. Thus, descriptions with generality should be avoided.

**(Response)**

**We agree with the reviewer to the point that Ghosal et al (2018) described a special case. Therefore, for clarity, we revised “The carp eDNA concentration increased 500× at night when the fish biomass only doubled [40]” as “Nocturnal carp eDNA concentrations increased 500-fold at night when fish biomass only doubled at a feeding site [36]” (Line 95-96).**

L157, 169: “-20 °C” → “−20 °C”

**(Response)**

**We revised them according to the suggestion (Lines 210).**

L182: “Each PCR reaction system included” → “Each PCR reaction included”

**(Response)**

**As per the reviewer’s suggestion, we revised “Each PCR reaction system included” as “Each PCR reaction included” (Line 236).**

L190: “There were three replicates per sample” → There were three replicates per sample “and standard DNAs”?

**(Response)**

**As per the reviewer’s suggestion, we added “and standard DNAs” after “There were three replicates each of all samples” (Lines 243-244).**

L198: “each target species” → each target individual ?

**(Response)**

**As per the reviewer’s suggestion, we revised “each target species” as “each target individual” (Line 252).**

L174-176, L423-426: I still have a question as to why we did not use other species-specific primers and probe sets designed in other regions. Again, the specificity is not so important in this study because tank water was used in all experiments.

In the responses to the reviewers, the authors accepted that D-loop is more likely to accumulate intraspecific genetic diversity than other regions. The other mitochondrial regions can also be polymorphed, but the "mutation rate" vary widely among regions. I agree that the presence of polymorphisms is an issue to be generally careful. However, in the eDNA study, the risk of underestimation caused by the primer/probe mismatch can be reduced by selecting regions that generally have lower mutation rates than D-loop.

In my opinion, in this manuscript, the authors should include the discussion about the selection of the target region to reduce the risk of mismatches and the reason why the primer-probe set designed in the D-loop was used in this study.

**(Response)**

**We thank the reviewer for this constructive comments. We added the discussion about the selection of the target region (D-loop) in the discussion section (Line 512-519).**

**This primers/probe set has been also used in Kasai et al. (2020) and Kasai. et al (2021), who conducted a nationwide eel distribution survey in Japan. Individual tank experiment of our study using this primers/probe sets revealed that polymorphism in the probe region strongly affected eDNA detection. We carefully checked the specificity in the set of primers, then we used this set in our study. It is necessary to design primers/probe set selecting lower intraspecific polymorphic regions, when eDNA concentration of each individuals must be precisely measured.**

**This point has been emphasized in the revised manuscript.**

L394-396: The eDNA concentration was different among samples. Thus, the comparison of Ct values here is meaningless.

**(Response)**

**As per the reviewer’s suggestion, we delated the sentence describing Ct values (Line 465-466).**

・In my opinion, in all tables and Figures, eDNA concentration should be shown per volume of filtered water (500 mL) or per PCR template (2 µL).

**(Response)**

**We consider this suggestion of the reviewer very carefully. As an independent experiment it is indeed sensible to express eDNA concentration per filtered water (500 mL) or PCR template (2** µL**). Meanwhile it seems to be common to express eDNA concentraiton in copies / L (Maruyama et al. 2014; Klobucar et al. 2017; Horiuchi et al. 2019; Yates et al. 2020 etc). Furthermore, the present study is strongly related to Kasai et al. (2021) in which the same primers-probe set was used to detected eel eDNA in natural waters of 50–600 mL per site then it was converted to be copies / L. Therefore we would like to be consistent to this expression.**

・Please check the following points: (1) Are coloured letters allowed in PLOS One? (2) Are the legends inserted in the correct place in the manuscript?

**(Response)**

**We thank the reviewer for pointing them out. (1) We revised coloured letters (Line 356, 361-363, 377-379, 404). (2) Submission Guidelines of PLOS ONE says “The (figure) caption may also include a legend as needed”, a newline before the legend was added in the figure caption example, and “Place legends, footnotes, and other text below the table”. It also says “You may also include a legend in your caption, but it is not required”. Therefore, we added newlines before the legends in the figures, and delated legends from tables and figures in the Supporting Information section (Line 808-848).**

Table1: Please add the Tm of the probes that are expected in each situation (Non variant, Vatiant-1, and Variant-2).

**(Response)**

**As we use MGB (minor groove binder) probe, it is not possible to calculate the expected Tm including the effect of mismatch nucleotides between the variants and the probe sequence due to the non-disclosure of calculation mechanism (the patent is held by ThermoFisher).**

Fig2-1(D): What does the Y-axis indicate?

**(Response)**

**We thank the reviewer for pointing out this flaw. Y-axis had been hiding behind. We brought it to the front.**

Fig2-1 and 2: To compare the eDNA concentration for each sample quantified by TaqMan (Fig. 2-1) and SYBR (Fig. 2-2), I recommend using sample-specific marks.

**(Response)**

**As per the reviewer’s suggestion, we revised the figures to have sample-specific marks; square: A-1; diamond: B-1; triangle: C-1; cross: A-2; bar: B-2; circle: C-2. Figure captions have also been revised accordingly (Fig2-1 and Fig2-2).**

Fig.2-1(A), 2(A): The red and orange dots are difficult to distinguish, so I recommend changing the colour.

**(Response)**

**As per the reviewer’s suggestion, we revised the colour of orange as green in Fig.2-1(A), Fig.2-2(A).**

S2 table: “between 5'-primer F and probe” → “between 3'-primer F and probe” ?

“between probe and primer R-3'” → “between probe and primer R-5'” ?

“*Green: base different from sample 0525_A-1.” → What does 0525 mean?

**(Response)**

**We revised “between 5'-primer F and probe” as “between primer F-3' and probe”, and “between probe and primer R-3'” as ”between probe and 3'-primer R”. “0525” means sampling day.**

7. PLOS authors have the option to publish the peer review history of their article (what does this mean?). If published, this will include your full peer review and any attached files.

If you choose “no”, your identity will remain anonymous but your review may still be made public.

Do you want your identity to be public for this peer review? For information about this choice, including consent withdrawal, please see our Privacy Policy.

Reviewer #1: No

[NOTE: If reviewer comments were submitted as an attachment file, they will be attached to this email and accessible via the submission site. Please log into your account, locate the manuscript record, and check for the action link "View Attachments". If this link does not appear, there are no attachment files.]

While revising your submission, please upload your figure files to the Preflight Analysis and Conversion Engine (PACE) digital diagnostic tool, https://pacev2.apexcovantage.com/. PACE helps ensure that figures meet PLOS requirements. To use PACE, you must first register as a user. Registration is free. Then, login and navigate to the UPLOAD tab, where you will find detailed instructions on how to use the tool. If you encounter any issues or have any questions when using PACE, please email PLOS at figures@plos.org. Please note that Supporting Information files do not need this step.

In compliance with data protection regulations, you may request that we remove your personal registration details at any time. (Remove my information/details). Please contact the publication office if you have any questions.
